# Quasielastic Neutron Scattering Study on Thermal Gelation in Aqueous Solution of Agarose

**DOI:** 10.3390/gels9110879

**Published:** 2023-11-06

**Authors:** Noriko Onoda-Yamamuro, Yasuhiro Inamura, Osamu Yamamuro

**Affiliations:** 1 Department of Natural Sciences, School of Science and Engineering, Tokyo Denki University, Hiki-gun, Saitama 350-0394, Japan; 2 Neutron Science Laboratory, Institute for Solid State Physics, University of Tokyo, 5-1-5 Kashiwanoha, Kashiwa, Chiba 277-8581, Japan; yasuhiro.inamura@j-parc.jp (Y.I.); yamamuro@issp.u-tokyo.ac.jp (O.Y.)

**Keywords:** agarose, dynamics, quasielastic neutron scattering, QENS, thermal gelation

## Abstract

The dynamics of water and agarose molecules in an agarose aqueous solution has been studied by means of quasielastic neutron scattering (QENS). The dynamic structure factor *S* (*Q*,*E*) of the agarose aqueous solution was fitted well to the sum of the Lorentz and delta function. The former is attributed to the diffusive motion of water molecules and the latter to the local vibrational motion of agarose molecules. The self-diffusion coefficient *D* of water molecules was obtained from the *Q*-dependence of the width of the Lorentz function, while the mean square displacement <*u*^2^> of agarose molecules was obtained from the *Q*-dependence of the intensity of the delta term. In the cooling direction, both *D* and <*u*^2^> decreased with decreasing temperature and showed discontinuous changes around the thermal gelation temperature (around 314 K). In the heating direction, however, *D* and <*u*^2^> did not show the obvious change below 343 K, indicating a large hysteresis effect. The present results of <*u*^2^> and *D* revealed that the thermal gelation suppresses the motion of the polymer and accelerates the diffusion of water molecules. The activation energy *E*_a_ of the diffusion of water in the sol state is the same as that of bulk water, but the *E*_a_ in the gel state is clearly smaller than that of bulk water.

## 1. Introduction

Polymer gels are those in which polymers are cross-linked to form a three-dimensional network structure that absorbs the solvent inside and swells. They are classified according to their bonding mode into chemical gels and physical gels, with the former being cross-linked by covalent bonds and the latter by various non-covalent interactions. The thermally reversible gelation that occurs in the physical gel of polymer hydrogels is driven by the coordination of various intermolecular interactions. The gelation happens on heating or cooling depending on the interactions. Among polysaccharides, methylcellulose gels are formed on heating owing to hydrophobic interactions and agarose gels on cooling owing to hydrogen bond formation. *κ*-Carrageenan gels are formed on cooling owing to hydrogen bond formation and week electrostatic interaction. Macroscopic properties change drastically with gelation. We are interested in how microscopic motions, such as the diffusive motion of water molecules and the segmental motion of polymer molecules, also change associated with gelation. We have studied the dynamics of water and polymer molecules in methylcellulose aqueous solutions using a quasielastic neutron scattering (QENS) technique and revealed that the microscopic motions of both water and polymer molecules give rise to dynamical slowing down on thermal gelation [1].

**Figure 1:**
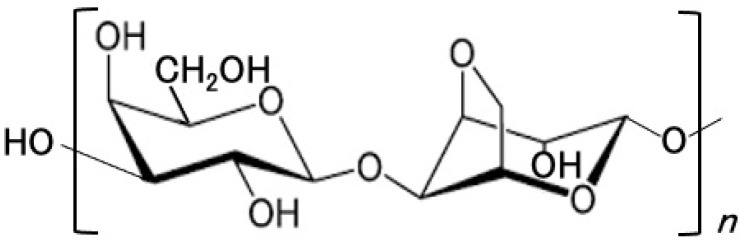
Chemical structure of agarose.

In this study, we focused on agarose. Agarose is a natural neutral polysaccharide whose ideal repeating units are (1,4)-linked 3,6-anhydro-α-L-galactose and (1,3)-linked β-D-galactose [2], as shown in Figure 1, and has long been used as a food additive. 

It is insoluble in water at room temperature, but it dissolves at higher temperatures (80–95 °C) and gels on cooling. Agarose is widely used in other fields besides food, such as culture media for micro-organisms and cells, electrophoresis gels for the analysis of proteins and nucleic acids, and carriers for gel permeation chromatography, due to its non-toxic nature, the temperature range of gelation being close to room temperature, the large hysteresis (difference between gelation temperature and melting temperature) and the neutrality of the gel without any ionic groups. Many physical and chemical properties have been reported using mechanical [3,4,5,6,7,8], rheological [3,4,5,9,10], thermal [3,4,5], scattering [4,9,11,12,13] and NMR [14,15] techniques. It is recognized that agarose molecules are randomly coiled in solution (sol), but on cooling, they form helices, which subsequently aggregate to form a three-dimensional cross-linked structure, and the system becomes a gel [16]. However, there is disagreement as to whether the higher-order structure is made up of a single helix [11,12] or a double helix [13]. It is also commonly accepted that gelation of agarose occurs through a liquid–liquid phase separation. However, whether the mechanism is due to spinodal decomposition [17,18] or nucleation-growth [19] remains to be elucidated. Others report that the sol–gel transition is a transition in polymer connectivity, while phase separation is due to thermodynamic instability, and that the two are different mechanisms [20].

The purpose of this study is to investigate the dynamics of water molecules and polymer molecules in aqueous agarose solutions using quasi-elastic neutron scattering (QENS) and to determine how the microscopic motions of water molecules and polymer molecules change during thermal gelation. The agarose solution becomes turbid with gelation and exhibits considerable hysteresis between gelation and melting temperatures [10]. Prior to the QENS experiment, the transmittance of visible light was measured to determine the gelation temperature and hysteresis of the sample solutions.

## 2. Results and Discussion

### 2.1. Transmittance of Visible Light

**Figure 2:**
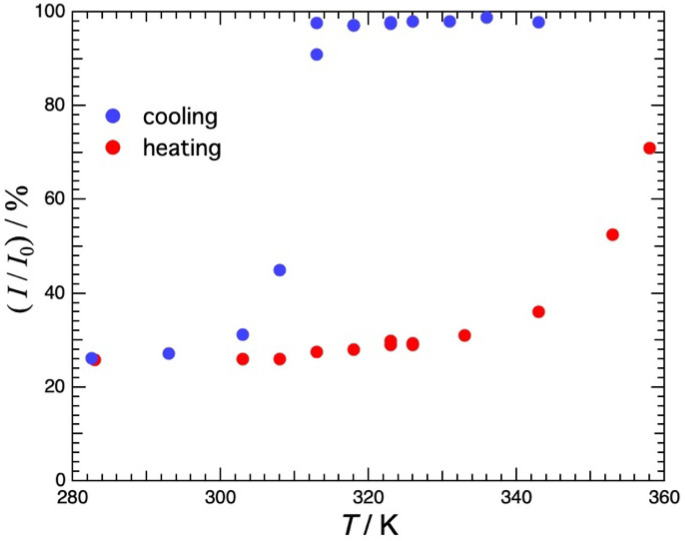
Temperature dependence of the transmittance of visible light at λ = 600 nm in agarose 3.0% H_2_O solution. Measurements were first taken in the cooling direction and then in the heating direction. Large hysteresis with a temperature range exceeding 40 K was observed.

Figure 2 shows the temperature dependence of the transmittance of visible light at λ = 600 nm in agarose 3.0% H_2_O solution. The data were taken in the cooling direction and then in the heating direction. On cooling, the transmittance is almost constant down to 313 K, decreases abruptly at 313 K and is constant below 300 K. The cloudiness should be due to the spinodal decomposition or first-order phase separation accompanied by the sol–gel transition [17]. In the subsequent heating from 282 K, the transmittance increases slightly up to around 340 K and then increases sharply thereafter. At 360 K, however, only about 80% of the transmittance before cooling is recovered. The temperature range of hysteresis was found to be large, exceeding 40 K.

### 2.2. Quasielastic Neutron Scattering (QENS)

**Figure 3:**
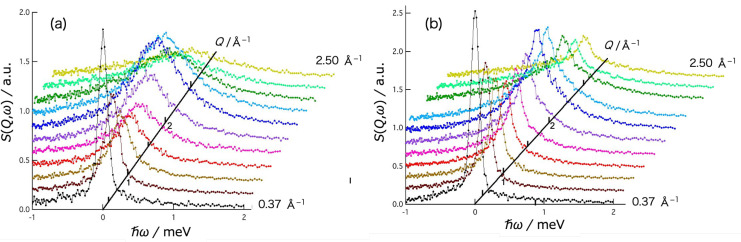
*Q*-dependence of dynamic structure factor *S*(*Q*,*ω*) of (**a**) D_2_O and (**b**) agarose 2.8% D_2_O solution obtained at 310 K. The values on the *Q* axis correspond to the ℏω = 0 position in the spectrum (*Q* depends on energy transfer in the time-of-flight method).

Figure 3 shows the *Q* dependence of the dynamic structure factor, *S*(*Q*,*ω*), of the water (D_2_O) (a) and the agarose aqueous solution (b) at *T* = 310 K. The *S*(*Q,ω*) for both water and agarose solution exhibit apparent *Q* dependent broadening of the scattering peak, plus keeping a part of the sharp elastic peak in agarose solution. The data of water fitted well to the Lorentz function *L*(*ω*) and the data of agarose solution to the sum of the Lorentz function *L*(*ω*) and the delta function *δ*(*ω*), both of which are convoluted by the resolution function *R*(*Q*,*ω*) of AGNES spectrometer. The fitting function used for the solution data is

*S*(*Q*,*ω*) = *R*(*Q*,*ω*)⊗[*A*_D_(*Q*)*δ*(*ω*) + *A*_L_(*Q*)*L(ω)*] + BG (1)

(2) $$L\left(\omega \right)=\frac{1}{\pi }\frac{\Gamma }{\omega^{2}+\Gamma^{2}}$$

where BG is a flat background, *A*_D_ and *A*_L_ are the fractional intensity for the delta and Lorenz functions, respectively, Γ is a half-width at half-maximum (HWHM), and the symbol ⊗ represents the operator of the convolution. In the *Q* range (0.26–2.70 Å^−1^) and energy resolution (0.12 meV) of AGNES, the relaxation and diffusive motions in the time domain of 0.1–10 ps appear as quasi-elastic scattering, so that the Lorentz component is attributed to the diffusive motion of water molecules and the delta component to the local vibrational motion of agarose molecule.

**Figure 4:**
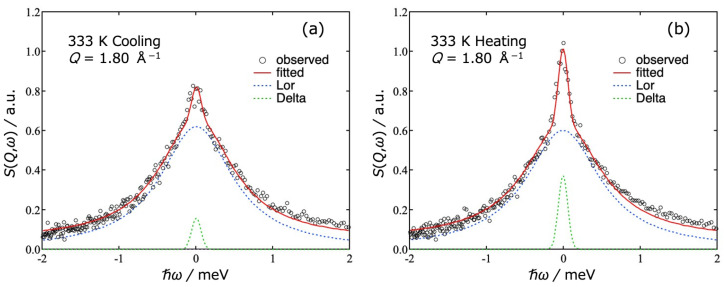
Dynamic structure factor *S*(*Q*,*ω*) of agarose solution observed at *Q* = 1.8 Å^−1^ and *T* = 333 K in the cooling (**a**) and heating (**b**) runs. The open circles are experimental data. The curves are the results of the fitting to Equation (1). The green, blue, and red curves represent delta part, Lorentz part and the total of fitting curves, respectively.

Figure 4 shows typical *S*(*Q*,*ω*) data observed at *Q* = 1.80 Å^−1^ and *T* = 333 K in the cooling direction (a) and the heating direction (b). The green, blue, and red curves represent the delta part, Lorentz part and the total of fitting curves, respectively. The data were taken in the cooling direction and then in the heating direction. At 333 K, the sample is in a sol state during the cooling process and in a gel state during the heating process. With increasing *Q*, the Γ values of the Lorentz function increased and the peak intensity of the delta term decreased.

**Figure 5:**
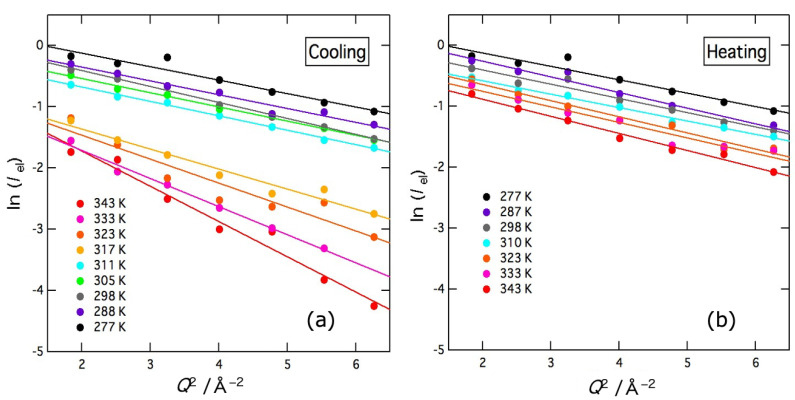
*Q*^2^ dependence of the elastic intensity (delta term of the fitting function) of agarose solution at various temperatures on cooling (**a**) and heating (**b**). Measurements were first taken in the cooling direction and then in the heating direction. Solid lines represent the results of the fit to Equation (3).

The elastic intensity *I*_el_ of the delta term determined by the fitting is plotted in a logarithmic scale as a function of *Q*^2^ in Figure 5. The figures show the results in the cooling direction (a) and heating direction (b), respectively. In the cooling direction, the elastic intensity shows a sharp increase between 317 K and 311 K, while no similar change is observed in the heating direction. When harmonic oscillators are assumed to represent the thermal motion of molecules, the elastic intensity depends linearly on temperature on a logarithmic scale. Therefore, the abrupt change in the cooling direction should be due to gelation, but the reverse transition does not occur in the heating direction owing to a large hysteresis effect. This result is consistent with the transmittance ones shown in Section 2.1. For a harmonic oscillator, the elastic intensity *I*_el_ is given by

*I*_el_ ∝ exp{−<*u*^2^>*Q*^2^} (3)

where <*u*^2^> denotes the mean-square displacement. As clearly shown in Figure 5, the data of ln (*I*_el_) were fitted well by straight lines and <*u*^2^> were evaluated as a function of temperature. In this fitting, the data below 1.2 Å^−1^ were omitted since the Lorentz function with a narrow width interferes with the delta function. In the cooling direction, there is a clear gap in ln (*I*_el_) intensity and slope between 317 K and 311 K. In the heating direction, however, the temperature dependence was small and continuous.

**Figure 6:**
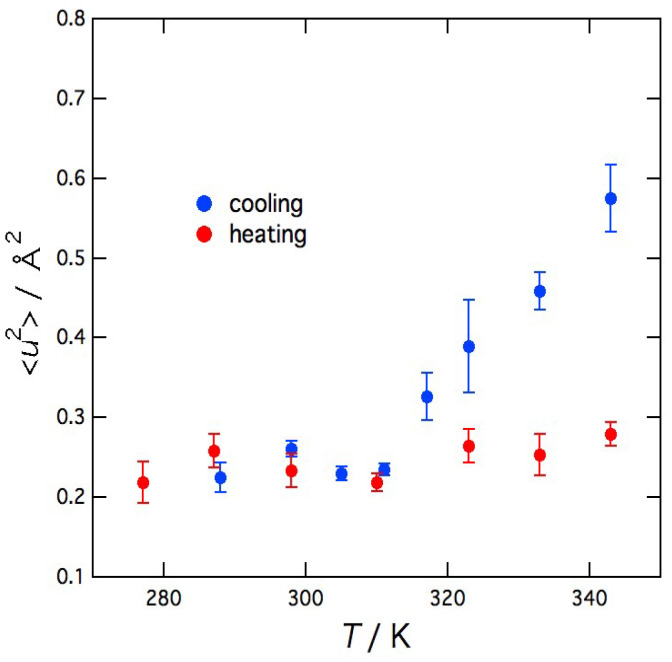
Temperature dependence of the mean-square displacement <*u*^2^> of the local vibrational motion of agarose molecules.

The temperature dependence of the mean-square displacement <*u*^2^> of the local vibrational motion of agarose molecules, which was obtained from Equation (3), is shown in Figure 6. In the cooling direction, <*u*^2^> decreases steeply with decreasing temperature but suddenly becomes constant below 311 K. In the heating direction, <*u*^2^> remains almost constant or only slightly increases with increasing temperature. These results show that <*u*^2^>, which is microscopic information, is related to the macroscopic phenomena of the sol–gel transition such as the changes in light transmittance and viscosity. 

In the *Q* region lower than the position of the first sharp diffraction peak of D_2_O (ca. 1.9 Å^−1^), the incoherent scattering from *D* atoms (*σ*_inc_(D) = 2.0 barns) is dominant. For a simple diffusion process, therefore, the half width at half maximum Γ values of the Lorentz function are reproduced by

**Figure 7:**
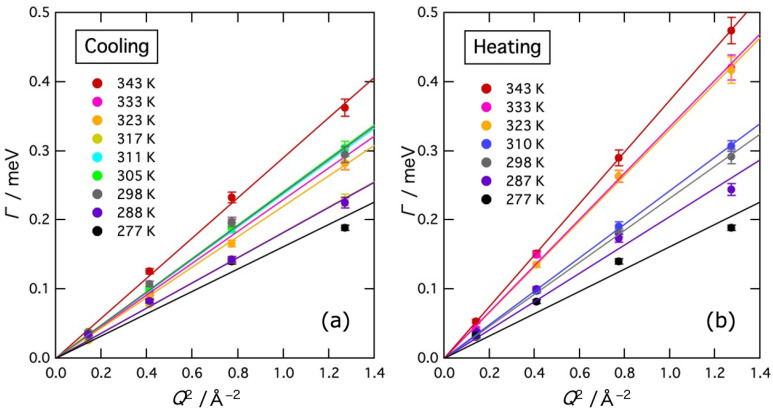
*Q*^2^ dependence of the half width at half maximum Γ values of the Lorentz function at various temperatures on cooling (**a**) and heating (**b**). Solid lines represent the results of the fit to Equation (4).

Γ = *DQ*^2^ (4)

where *D* is the self-diffusion coefficient of water molecules [21]. Figure 7 shows Γ values plotted as a function of *Q*^2^ in the region *Q* < 1.2 Å^−1^. As mentioned earlier, interference between the Lorentz and delta components occurred in the region of *Q* < 1.2 Å^−1^ in the fitting, so in this region, the intensity of the delta component was determined by extrapolating the high *Q* data with a straight line as shown in Figure 5, and then, Γ was obtained by the fitting. The Γ data were actually fitted well by straight lines passing through the origin. In the higher *Q* region, it is expected that the coherent scattering from water molecules becomes important and Γ exhibits complicated phenomena such as De Gennes narrowing [22]. In this experiment, however, De Gennes narrowing was not detectable technically because the determination of the Lorentz term is not reliable due to the relatively larger delta term at higher *Q* regions. 

**Figure 8:**
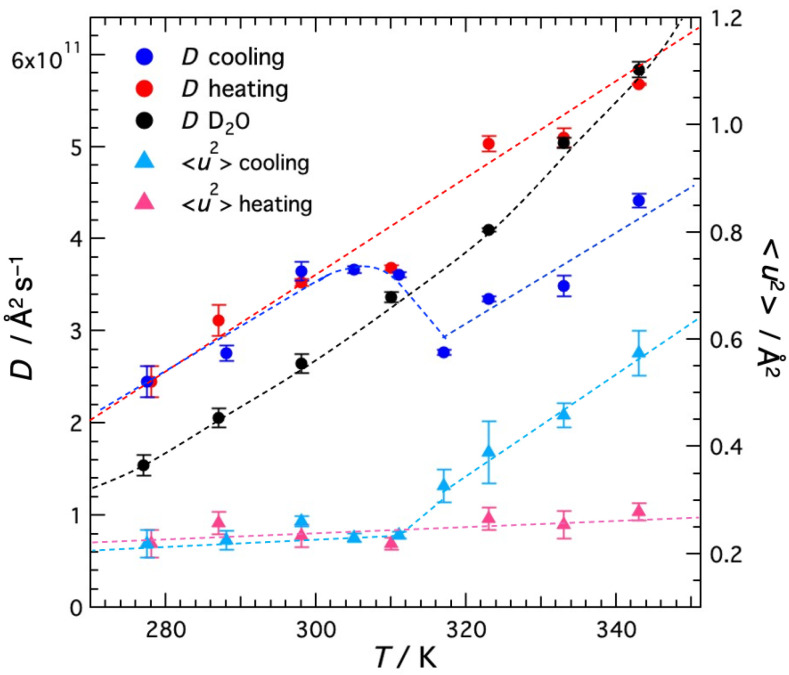
Temperature dependence of the self-diffusion coefficient *D* for D_2_O molecules in agarose solution and bulk D_2_O. The mean-square displacement <*u*^2^> of the local vibrational motion of agarose molecules shown in Figure 7 are redisplayed for reference. Dashed curves are the guides to the eye.

The temperature dependence of the self-diffusion coefficient *D* for water molecules in agarose solution and that for bulk water are shown in Figure 8, along with <*u*^2^> plotted in Figure 6. In the cooling direction, *D* decreases with decreasing temperature, but abruptly increases around 310 K and decreases again below 300 K. In the heating direction, *D* monotonously increases with increasing temperature up to 343 K, the upper limit of the measured temperature. The large hysteresis, that is sudden gelation occurring around 310 K in the cooling direction and no melting even at 343 K in the heating direction, is consistent with the visible light transmission Section 2.1 shown earlier.

The sudden increase in *D* of water is thought to be due to the two effects occurring for agarose; (1) agarose helices aggregate to form a three-dimensional network during gelation and (2) solute agarose is incorporated into the network. These effects decrease the agarose concentration, reduce the diffusion resistance, and promote water diffusion. This result is consistent with the NMR work by Dai et al. [15]. They performed pulsed magnetic field-gradient stimulated echo (PGSTE) ^1^H NMR experiments on aqueous agarose solutions with dendrimers as probe molecules and reported that the diffusion coefficient of dendrimers increased with gelation. They explained that the network formation of agarose aggregated bundles and the decrease in solute agarose concentration reduced the hydrodynamic drag and enhanced the diffusion of probe molecules [15]. The present results directly, without using the probe molecules, demonstrate that gelation promotes the diffusion of water molecules. Thus, the results of <*u*^2^> and *D* in the cooling direction revealed that thermal gelation significantly changes the microscopic motion of both the polymer and water; the polymer slows down and the water accelerates. 

It is also worth noting that the *D* of water in the gel is greater than the *D* of bulk water. The same case has been reported in other systems. Suzuki’s group investigated the hydration properties of actin filaments (F-actin) [23,24,25] and adenosine phosphate ion (ATP) [25,26] by using high-resolution microwave dielectric spectroscopy. They reported that these solutes are surrounded by fast water molecules (Hyper Mobile Water HMW) with much higher mobility than bulk water molecules, as well as typical hydrated water molecules with low rotational mobility. In addition, the rotational degrees of freedom of water molecules around charged particles and the dielectric relaxation process of water molecules around ions are explained by two theoretical approaches [25]. The presence of HMW was not observed in myoglobin, a globular protein. From these, they concluded that the large surface charge density and structure (shape) of the molecule induce HMW. Yamada et al. [27] investigated the dynamical behavior of hydration water sandwiched between 1,2-dimyristyl-sn-glycero-3-phosphocholine (DMPC) bilayers using quasi-elastic neutron scattering (QENS) The QENS profile of hydrated water was expressed as the sum of three Lorenz functions. They are attributed to (1) free water with a diffusion coefficient (D) comparable to that of bulk water, (2) loosely bound water with D one order of magnitude smaller than free HW, and (3) tightly bound water with D comparable to that of DMPC molecules. Recently, Rahman et al. [28] reported QENS results for hydrated water incorporated between DMPE bilayers, which differ slightly in headgroup structure from DMPC. The QENS profile of hydrated water was expressed as the sum of three Lorentz functions as in DMPC. Two components, the free water and loosely bound water (although the diffusion of loosely bound water was slightly faster in DMPE than in DMPC), were similar to those in DMPC. However, no tightly bound hydrated water was observed, instead fast water, identified to rotational motion, was observed. The observed relaxation time of the rotational water between the DMPE bilayers was approximately six times faster than in DMPC. The authors cited the MD simulation by Higuchi et al. [29] that -NH_3_^+^ in the DMPE headgroup is more strongly bound to water than -N(CH_3_)_3_^+^ in the DMPC headgroup, and they considered that water molecules bound to -NH_3_^+^ will break hydrogen bonds with other water molecules and thus activate the movement of water around them. Furthermore, they concluded that the dynamics of water near the lipid headgroup depends on the charge of the lipid headgroup.

In the present study, the spectra obtained in the QENS experiment were divided into elastic components (delta components) and quasi-elastic components (Lorentz components). If the Lorentz components are further divided, it is expected that water with different kinetic rates, such as DMPC and DMPE, will be identified. However, considering the energy window of AGNES, which is narrower and higher than those of the spectrometers in the reported works, it is not possible to further divide the present data. AGNES is thought to preferentially observe the faster water relaxation (higher energy corresponds to faster motion). Thus, the obtained diffusion coefficient of water in gel is greater than that of bulk water, suggesting that fast water, similar to DMPE, is present in the agarose gel. Agarose is usually neutral and contains no ionic groups, but the agarose type IV (special high EEO) used in this study contains 0.04% sulfate. Thus, in aqueous agarose type IV solutions, the surface charge of the agarose molecules is as high as that of F-actin and DMPE, and the helical structure in the gel state, like the filament structure of F-actin, is thought to satisfy the shape factor that enhances the surface charge density.

**Figure 9:**
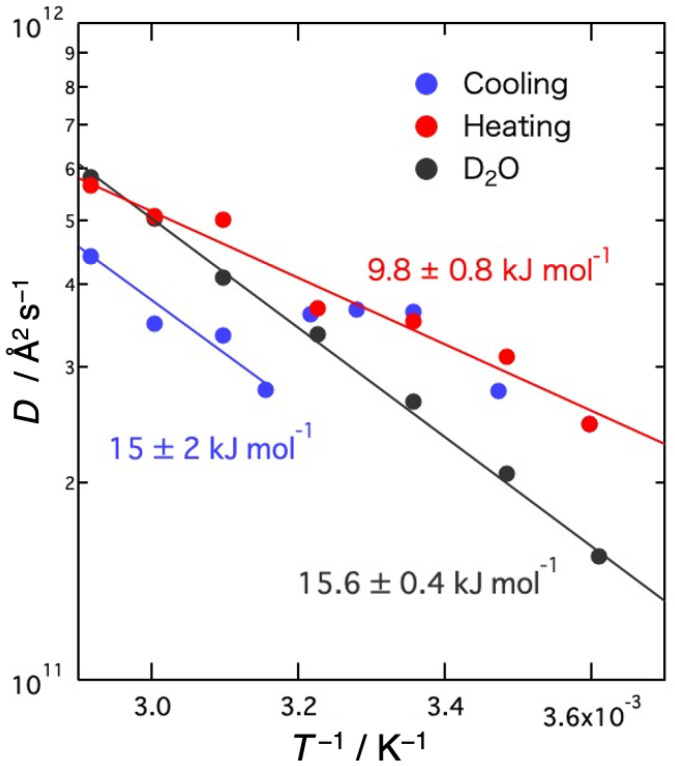
Arrhenius plots of the self-diffusion coefficients *D* of D_2_O molecules in agarose solution and bulk D_2_O. Solid lines represent the results of the fit with assuming the Arrhenius law. The numerical value is the activation energy corresponding to each line.

Figure 9 shows the Arrhenius plots of the self-diffusion coefficients *D*. Activation energies (*E*_a_) obtained from the slope of *D* are 15 ± 2 kJ mol^−1^ for the sol state and 9.8 ± 0.8 kJ mol^−1^ for the gel state, compared to 15.6 ± 0.4 kJ mol^−1^ for the bulk D_2_O. *E*_a_ is not different from bulk water in the sol state, but it is clearly smaller in the gel state. This result may be due to the distorted hydrogen bond structure of water between agarose networks compared to bulk water. In DMPE, where the existence of fast water has been confirmed, the activation energy of free water, whose diffusion coefficient is slightly faster than that of bulk water, is 4 kJ mol^−1^, which is clearly smaller than that of free water in DMPC bilayers (19 kJ mol^−1^), where the diffusion coefficient is comparable to that of bulk water, and that of bulk water [28]. Considering these results and the results of MD simulations [29], it has been suggested that the hydrogen bonds between free water molecules between DMPE bilayers may be more distorted than those between DMPC bilayers.

## 3. Conclusions

The microscopic dynamics of water molecules and agarose molecules in aqueous agarose solutions were investigated using the QENS method. The obtained dynamic structure factors were separated into Lorentz function and delta function components, and the former was analyzed due to the self-diffusion motion of water molecules and the latter due to the local vibrational motion of agarose molecules. Both the self-diffusion coefficient of water molecules and the mean square displacement of agarose molecules showed discontinuous changes with the sol–gel transition in the cooling direction. In the gel state, the motion of the hydration water is accelerated and that of the agarose polymer is suppressed. In the heating direction, neither changed discontinuously, indicating that no reverse transition occurred. This large temperature hysteresis was consistent with the prior transmittance of visible light results. The activation energy of the self-diffusion coefficient of water was about 15 kJ mol^−1^ in the sol state, comparable to that of bulk water, and about 10 kJ mol^−1^ in the gel state, clearly smaller than that of bulk water. The present QENS study has revealed the following microscopic picture of the sol–gel transition of agarose aqueous solutions. The gelation reduces <*u*^2^>, the local motion of polymer segments, due to the binding of polymer chains and the creation of a network. On the other hand, the motion of water molecules is accelerated due to the breaking of the local hydrogen-bonded structure, caused by the interaction with the helical chains to form a network structure via gelation.

## 4. Materials and Methods

### 4.1. Materials

The agarose used in this experiment was Sigma-Aldrich Type IV, Special high EEO type (A3643, sulfate content = 0.04%, EEO = 0.30%, gel strength = 1183 g cm^−2^, *T*_gel_ = 36.5 °C). The concentration of agarose in D_2_O solution was 2.8 wt% (3.0 wt% H_2_O solution corresponds to 2.8 wt% D_2_O solution if the molar ratio of agarose to water is kept the same). Agarose was vacuum dried before use and then dispersed in water in a screw-top bottle and stirred with a magnetic stirrer for about 5 h at room temperature, then slowly heated and stirred to 95 °C in a water bath and kept for about 30 min to prepare the sample. After this treatment, the agarose was completely dissolved. Transmittance was measured for H_2_O solution with 3.0% agarose concentration, and QENS was measured for D_2_O solution with 2.8% agarose concentration.

### 4.2. Quasielastic Neutron Scattering (QENS) Measurement

Neutron scattering data were collected with a high-resolution pulse cold-neutron spectrometer AGNES [30,31] belonging to Institute for Solid State Physics (ISSP), University of Tokyo. This spectrometer is installed with the cold neutron guide (C3-1) of the research reactor, JRR-3 in Japan Atomic Energy Agency (JAEA), Tokai, Japan. Neutrons with a wavelength of 0.422 nm are extracted with an array of five PG(002) monochromators and pulsed with a double Fermi chopper. The pulsed neutrons are scattered by a sample and detected with 328 He tube detectors arranged in a wide detector arranged in a wide detector bank covering the scattering angles of 10–130°. The energy resolution (full width at half-maximum of the elastic peak) of Δ*E* was 0.12 μeV. The accessible energy windows, *Q* = (4π/*λ*)sin*θ*, where 2*θ* denotes scattering angle and λ denotes the wavelength of incident neutrons) range of the spectrometer, are –3 meV< ℏω <20 meV, 0.2 < *Q* < 2.7 Å^−1^, respectively. The data of a sample solution at 168 K, where the motion of water molecules and agarose molecules is completely frozen, were used as the resolution function of the instrument. The measured time-of-flight spectra were normalized using the vanadium standard, corrected for transmission, subtracted from the contribution of the sample cell, and transformed into the energy spectra. The raw data were reduced using AGDAS 3, an in-house software at AGNES.

In this experiment, D_2_O and normal (non-deuterated) agarose were used to observe both dynamics of water and agarose molecules. The number ratio of agarose monomer to water is about 1:560, so the number ratio of H to D atoms is about 1:60. Since the ratio of incoherent scattering cross section of H atom to D atom is about 40:1, the relative scattering intensity from H atoms of agarose to that from D atoms of D_2_O is expected to be comparable in this sample solution.

About 3.3 cc of the solution, prepared at 95 °C as above, was loaded in a concentric double cylinder Al cell kept at 70 °C, quickly indium-sealed and set in a cryostat kept at 330 K. The thickness of the sample was 2 mm, which corresponds to 88% neutron transmission, to avoid multiple scattering. The temperature of the samples was controlled by placing them in top-loading closed cycle refrigerators. Measurements were taken in the cooling direction from 343 K to 277 K and then in the heating direction up to 343 K. After reaching the target temperature, a wait of 30 min was allowed, and then, the measurement was started. The duration of the measurement was about 6 h for each. The measurement to obtain the resolution function was performed at 168 K. The empty cell was measured at room temperature, and the data were used as background. Bulk D_2_O was also measured for comparison.

### 4.3. Transmittance of Visible Light

Transmittance of visible light in agarose solution was measured using a Shimazu Spectronic 20A spectrometer (Kyoto, Japan). The wavelength of the incident light was 600 nm. The sample solution in the sample tube with an inner diameter of 13 mm was transferred to a thermostatic bath, waited for about 10 min after reaching each target temperature and then quickly set in the spectrometer to measure transmittance. At the temperature where the sol–gel transition starts, the measurement was made again after 30 min. The measurements were carried out in the temperature range 280–360 K in the cooling direction followed by the heating direction.

## Data Availability

Not applicable. All data and materials are available on request from the corresponding author. The data are not publicly available due to ongoing research using a part of the data.
